# Increased risk for T cell autoreactivity to ß-cell antigens in the mice expressing the A^vy^ obesity-associated gene

**DOI:** 10.1038/s41598-019-38905-z

**Published:** 2019-03-12

**Authors:** Jing Yong, Jide Tian, Hoa Dang, Ting-Ting Wu, Mark A. Atkinson, Ren Sun, Daniel L. Kaufman

**Affiliations:** 1 Department of Molecular and Medical Pharmacology, University of California, Los Angeles, CA 90095-1735 United States; 2 Present Address: Sanford-Burnham-Prebys Medical Discovery Institute, La Jolla, CA 92037 United States; 3 Departments of Pathology and Paediatrics, University of Florida Diabetes Institute, Gainesville, FL 32610 United States

## Abstract

There has been considerable debate as to whether obesity can act as an accelerator of type 1 diabetes (T1D). We assessed this possibility using transgenic mice (MIP-TF mice) whose ß-cells express enhanced green fluorescent protein (EGFP). Infecting these mice with EGFP-expressing murine herpes virus-68 (MHV68-EGFP) caused occasional transient elevation in their blood glucose, peri-insulitis, and Th1 responses to EGFP which did not spread to other ß-cell antigens. We hypothesized that obesity-related systemic inflammation and ß-cell stress could exacerbate the MHV68-EGFP-induced ß-cell autoreactivity. We crossed MIP-TF mice with A^vy^ mice which develop obesity and provide models of metabolic disease alongside early stage T2D. Unlike their MIP-TF littermates, MHV68-EGFP–infected A^vy^/MIP-TF mice developed moderate intra-insulitis and transient hyperglycemia. MHV68-EGFP infection induced a more pronounced intra-insulitis in older, more obese, A^vy^/MIP-TF mice. Moreover, in MHV68-EGFP-infected A^vy^/MIP-TF mice, Th1 reactivity spread from EGFP to other ß-cell antigens. Thus, the spreading of autoreactivity among ß-cell antigens corresponded with the transition from peri-insulitis to intra-insulitis and occurred in obese A^vy^/MIP-TF mice but not lean MIP-TF mice. These observations are consistent with the notion that obesity-associated systemic inflammation and ß-cell stress lowers the threshold necessary for T cell autoreactivity to spread from EGFP to other ß-cell autoantigens.

## Introduction

Epidemiologic studies have demonstrated that the incidence of type 1 diabetes (T1D) has been increasing globally over the past few decades, but the underlying reasons for this increase are not well understood^1,2^. It has been hypothesized that increases in childhood obesity may contribute in part to this rising incidence^3,4^. Obesity is associated with insulin resistance, low-grade chronic inflammation, higher levels of circulating inflammatory factors including cytokines and chemokines, altered ß-cell antigen presentation, and antigen presenting cell (APC) activation (reviewed in)^5^. In that context, naïve ß-cell-reactive T cells that previously ignored their cognate self-antigens might receive sufficient co-stimulation to activate. In some individuals with genotypes and environmental exposures that increase their susceptibility for developing T1D, these autoreactive T cells may be able to further expand and amplify ß-cell autoreactivity through epitope spreading^6,7^.

Multiple studies that have sought to identify a link between body mass and the development of ß-cell autoantibodies with subsequent T1D, with results having either supported or argued against an association (e.g.,)^8–18^. Those studies did not, however, monitor autoreactive T cells. Conceivably, T cell autoreactivity to ß-cell antigens may occur more frequently in obese individuals, perhaps only transiently and without inducing ß-cell autoantibodies. Such autoreactive T cell responses may, however, raise the risk for more robust T cell autoimmunity against ß-cells when other genetic and environmental T1D susceptibility factors are also present. Hence, we sought to experimentally test whether obesity could increase the propensity for developing T cell autoreactivity to ß-cells using new mouse models and a highly sensitive ELISPOT assay to detect low-frequency activated antigen-specific T cells.

Previous studies of transgenic mice expressing a foreign protein in their ß-cells demonstrated that their immune systems ignore ß-cells expressing the transgene-encoded protein^19–22^. However, when these mice were infected with a virus that expresses the transgene-encoded protein, ß-cell destruction could ensue, depending on the infectious agent, the particular ectopically expressed protein, the extent of T cell tolerance to that protein, and the animal’s genetic background^19–22^. To our knowledge, no study has asked whether obesity might exacerbate the animal’s response to virally-induced ß-cell neo-autoreactivity.

The mouse models studied herein possess a transgene in which a mouse insulin promoter (MIP) drives the expression of green fluorescent protein (GFP) or enhanced GFP (EGFP) specifically in their ß-cells. A wide range of viruses have been constructed to express EGFP in order to facilitate studies of their tropism and life cycle. Specifically, we were initially interested whether infection with a recombinant adenovirus, lymphocytic choriomeningitis virus (LCMV) or murine gammaherpesvirus-68 virus (MHV68) that directed the expression of EGFP could break self-tolerance to GFP in “MIP-GFP” mice that express GFP in their ß-cells^23^, and if so, would this neo-autoreactivity spread to other ß-cell antigens, promoting insulitis and T1D?

In later studies, we studied C57BL/6 “MIP-TF” mice that possess a transgene consisting of a mouse insulin promoter (MIP) linked to a trifusion (TF) protein of three linked imaging reporters; luciferase (for noninvasive charge-coupled device (CCD) imaging), a modified herpes virus thymidine kinase (for noninvasive microPET imaging) and EGFP (for fluorescent microscopy) of pancreatic islet ß-cells^24^. These mice express the trifusion reporter specifically in their ß-cells and offer the opportunity to make non-invasive longitudinal assessments of ß-cells using CCD, as in our past studies of these mice with STZ-induced T1D or high fat diet-induced T2D^24^.

We observed that infecting MIP-GFP mice with a mouse herpes virus (MHV68) that expresses EGFP (MHV68-EGFP)^25,26^ led to occasional peri-insulitis but no T cell autoimmunity to ß-cell antigens other than EGFP, and no hyperglycemia. We hypothesized that if the MIP-TF mice were made to be obese, the ensuing ß-cell metabolic stress and systemic chronic inflammation might increase the pathogenic potential of the autoreactive T cell response to EGFP following MHV68-EGFP infection. Mice possessing a dominant viable yellow (A^vy^) mutation spontaneously develop obesity, insulin resistance, and have been widely studied as models of metabolic disease as well as early stage T2D^27–33^. The A^vy^ mutation causes ectopic expression of the agouti signaling protein which is a potent antagonist of melanocortin-4 (Mc4r) receptors on hypothalamic neurons that exert an inhibitory effect on feeding behavior^31,33–35^. We crossed C57BL/6 MIP-TF mice with C57BL/6 A^vy^ mice to determine whether MHV68-EGFP infection of the resulting obese MIP-TF A^vy^ mice, as compared to their nonobese MIP-TF littermates, would lead to intra-insulitis and spreading of T cell autoreactivity from EGFP to other ß-cell antigens. The resulting observations provide experimental data with a well-defined system that are consistent with the notion that obesity-associated inflammation and ß-cell stress can exacerbate ß-cell autoimmunity. Other factors which may have also played a role in our observations are also discussed.

## Results

### MHV68-EGFP infection, but not adeno-EGFP or lenti-GFP infection, leads to mild peri-insulitis in mice that express EGFP in their ß-cells

**Figure 1:**
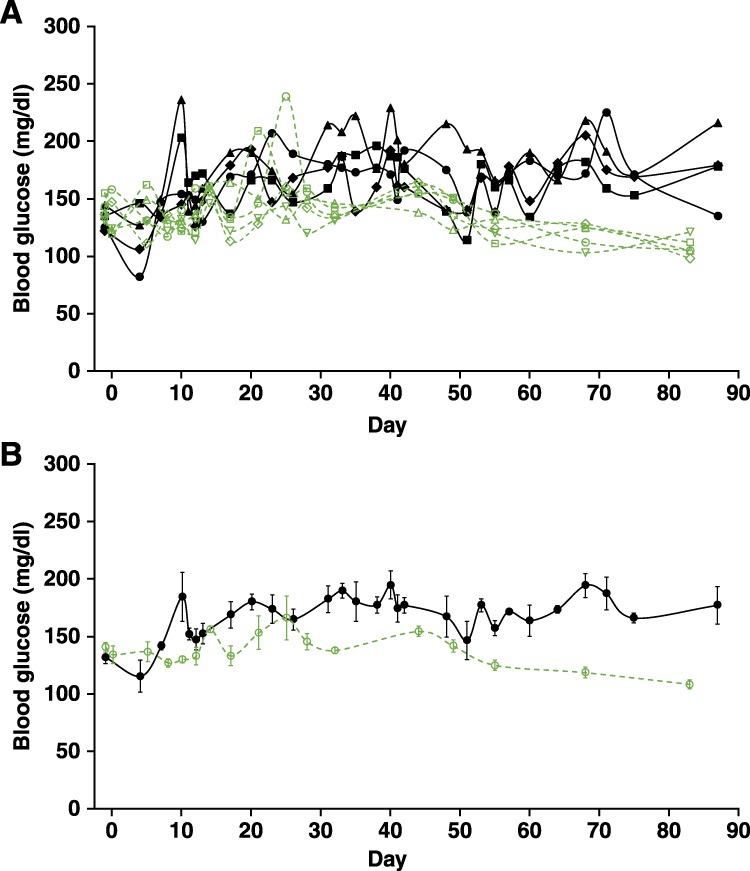
MHV68-EGFP infection induces a slight elevation in the blood glucose of some MIP-GFP mice. (**A**) C57Bl6 MIP-GFP mice and wildtype C57Bl/6 mice were infected with MHV68-EGFP and their blood glucose monitored longitudinally. Beginning about 10 days post-infection, blood glucose levels were on average, higher in MIP-GFP mice (black lines connecting solid symbols show blood glucose levels of individual mice, n = 4) compared to that in wildtype mice (green dashed lines connecting open symbols, n = 5). (**B**) Average blood glucose level over time for each group ± SEM.

In pilot studies, we infected twelve-week old C57BL/6 MIP-GFP mice with adeno-EGFP, LCMV-GFP, or MHV68-EGFP and monitored their blood glucose for 8–12 weeks post-infection. Infection with adeno-GFP or LVMV-EGFP did not lead to any obvious abnormalities in their blood glucose levels during the observation period (data not shown), and those viruses were not studied further. We did observe, however, an elevation in the blood glucose levels of some MHV68-EGFP infected mice beginning about day 10 post-infection, although this did not reach the level of overt hyperglycemia (>250 mg/dL, Fig. 1). We next infected a group of wildtype C57BL/6 mice with MHV68-EGFP and observed that on average, the blood glucose levels were lower in this group compared to that in MHV68-EGFP-infected MIP-GFP mice (Fig. 1). These data are consistent with the notion that the virally directed expression EGFP generates immune responses that cause dysregulation of ß-cells in MIP-GFP mice but have no apparent effect on the ß-cells of wildtype mice.

**Figure 2:**
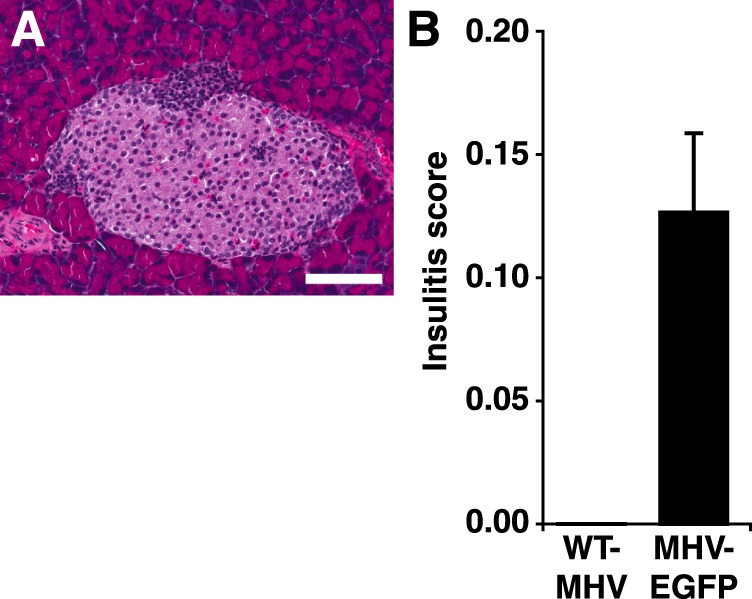
MHV68-EGFP infection, but not wildtype MHV68 infection, induces slight peri-insulitis in some islets of MIP-TF mice. (**A**) Representative image of peri-insulitis in a MHV68-EGFP-infected MIP-TF mouse at day 21 post-infection. White bar indicates 100 μm. (**B**) Insulitis was scored at 21 days-post-infection on a scale of 0–3, as described in Methods. Black bar = MHV68-EGFP infected (n = 8). Insulitis in wildtype MHV68-infected mice was at base-line (n = 5).

In subsequent studies, we switched from studying MIP-GFP mice to studying MIP-TF mice because the MIP-TF mice not only express EGFP in their ß-cells, but also luciferase which provides the opportunity to monitor their ß-cells noninvasively using CCD imaging. We examined the outcome of infecting MIP-TF mice with wildtype MHV68 versus MHV68-EGFP. In this study, infection with MHV68 or MHV68-EGFP led to neither elevated blood glucose levels, nor any significant differences in their body weight or bioluminescent signals from their pancreas region during a two-month observation period (data not shown). Infection with these viruses did not cause any abnormalities in glucose tolerance as measured by intraperitoneal glucose tolerance tests before and after infection (data not shown). We examined their pancreata for infiltrates at day 14, 21, 42 and 60 post-infection and scored the extent of insulitis at 21 days post-infection. We did not observe any islet infiltrates in mice that had received wildtype MHV68. In contrast, in mice that received MHV68-EGFP, a small portion of islets had mild peri-insulitis leading to an average insulitis score of 0.13 on a scale of 0–3 (Fig. 2). Thus, in the MIP-TF model, MHV68-EGFP infection did not cause obvious changes in the animal’s blood glucose or ß-cell bioluminescence, but at the histological level, the MHV68-EGFP (but not wildtype MHV68) infection led to mild to moderate peri-insulitis. Thus, MHV68-EGFP expression of EGFP induces low-grade autoreactivity to EGFP-expressing ß-cells.

### MHV68-EGFP infection induces low-grade intra-insulitis in young mildly obese A^vy^/MIP-TF mice

**Figure 3:**
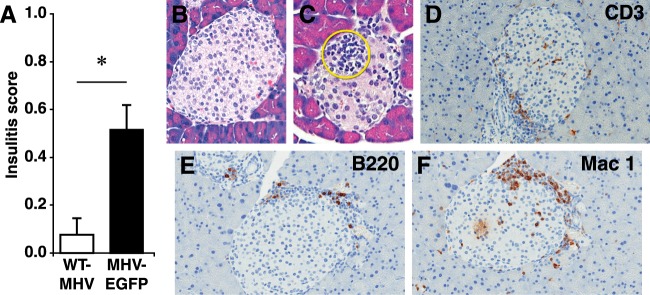
MHV68-EGFP infection leads to widespread intra-insulitis in young A^vy^/MIP-TF mice. (**A**) A^vy^/MIP-TF mice were infected at 10–12 weeks in age with wildtype MHV68 or MHV68-EGFP and twenty one days later their pancreata were examined for infiltrates. Eighty islets were scored from each animal. Bars show mean insulitis score in WT-MHV68 infected (open bar, n = 4) and MHV68-EGFP (black bar, n = 9) infected A^vy^/MIP-TF mice ± SEM, *p = 0.05). MHV68-EGFP infected A^vy^/MIP-TF mice also had significantly higher insulitis score than MHV68-EGFP infected MIP-TF mice (see Fig. 2, p < 0.01). Insulitis in control uninfected A^vy^/MIP-TF mouse pancreata was at base-line (not shown). Represenataive image of a WT-MHV68 infected (**B**) and MHV68-EGFP-infected (**C**) A^vy^/MIP-TF mouse pancreas section stained with H&E. Mononuclear cell infiltrates are circled in yellow. (**D**) Anti-CD3, (**E**) Anti-B220, and (**F**) Mac1 stained MHV68-EGFP-infected A^vy^/MIP-TF mouse pancreas sections.

We hypothesized that obesity may provide systemic inflammatory signals and/or ß-cell stress that alters ß-cell antigen presentation, such that an experimentally induced T cell response against EGFP could spread to other ß-cell antigens and promote insulitis and T1D. We crossed MIP-TF C57BL/6 mice that were heterozygous for the transgene with A^vy^/a C57BL/6 mice to obtain wildtype, MIP-TF, A^vy^, and A^vy^/MIP-TF littermates. As expected, mice carrying the A^vy^ gene began to develop obesity at 6–8 weeks of age. We infected 10–12-week-old MIP-TF, A^vy^ or A^vy^/MIP-TF mice with wildtype MHV68 or MHV68-EGFP. After 21 days, none of the infected mice had become hyperglycemic and their pancreases were examined for insulitis. As observed previously, MIP-TF mice infected with wildtype MHV68 virus had no discernable insulitis, but those infected with MHV68-EGFP had occasional mild peri-insulitis. A^vy^/MIP-TF mice that were infected with wildtype MHV68 had a very slight insulitis (average score of 0.08, Fig. 3A,B), which was related to a generalized low-grade pancreatitis in two of the four mice. These data suggest that MHV68 infection itself did not specifically induce insulitis in these mice. In contrast, A^vy^/MIP-TF mice that were infected with MHV68-EGFP developed a range of peri-insulitis to mild intra-insulitis in islets throughout the pancreas (Fig. 3C) leading to an average insulitis score of 0.52 (Fig. 3A, p < 0.05 vs. wildtype MHV68 infection, p < 0.01 vs MHV68-EGFP-infected MIP-TF mice). While the extent of insulitis was modest, the differences in insulitis between control and experimental groups were obvious and did not vary greatly among individual mice. None of the nine MHV68-EGFP infected A^vy^/MIP-TF mice developed pancreatitis.

We immunohistochemically examined the nature of the islet infiltrates in MHV68-EGFP-infected A^vy^/MIP-TF mice. We found CD3^+^ lymphocytes (Fig. 3D), B cells (Fig. 3E) and macrophages (Fig. 3F). Thus, the islet infiltrates have a composition similar to those observed in prediabetic NOD mice and humans with T1D^36^.

### MHV68-EGFP infection induces a more severe intra-insulitis in older more obese A^vy^/MIP-TF mice

**Figure 4:**
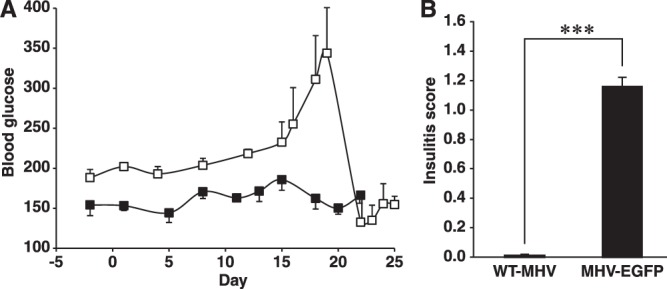
MHV68-EGFP infection causes transient hyperglycemia and more severe intra-insulitis in older A^vy^/MIP-TF mice. (**A**) Longitudinal blood glucose levels of A^vy^/MIP-TF mice after wildtype MHV68 (solid black square) or MHV68-EGFP (open square) infection. Data shown is mean blood glucose +/−SEM (n = 3–4 mice/group). (**B**) A^vy^/MIP-TF mice (25–30-week old) were infected with MHV68-EGFP or with wildtype MHV68. Eighty islets were examined and scored from each animal (n = 3 mice/group, ***p < 0.001).

We hypothesized that older A^vy^/MIP-TF mice, which have greater obesity and have had chronic inflammation as well as ß-cell stress for a longer period, may be more prone to developing insulitis after MHV68-EGFP infection. We infected 25–30 weeks old A^vy^/MIP-TF mice with wildtype MHV68 or MHV68-EGFP. Sixteen days after infection, some of the MHV68-EGFP-infected mice developed transient hyperglycemia (Fig. 4A). Their pancreases were examined 24 days post-infection. While mice infected with wildtype MHV68 had essentially no insulitis, those infected with MHV68-EGFP had an average insulitis score of 1.14 out of 3 (Fig. 4B), significantly greater than when A^vy^/MIP-TF mice received the same treatment at 10–12 weeks in age (Fig. 3A, p < 0.01). None of them developed pancreatitis.

### MHV68-EGFP-induced Th1-type autoreactivity spreads from EGFP to other ß-cell antigens in obese A^vy^/MIP-TF mice, but not in lean MIP-TF mice

The above histological studies do not inform whether the host’s the autoimmune responses following MHV68-EGFP infection were limited to the virally encoded EGFP or spread to other ß-cell antigens. To obtain a more detailed information on the extent of immune tolerance to ß-cell antigens, we used an ELISPOT assay capable of detecting single activated antigen-specific T cells. While insulin B-chain_(9–23)_, GAD65, HSPp277, and IGRP become targets of autoimmunity in NOD mice (K^d^, D^b^, I-E, and I-A^g7^), these antigens may not be the targets of ß-cell reactive T cells in the context of the H-2^b^ of MIP-TF and A^vy^/MIP-TF C57BL/6 mice. To cast a broad net to detect T cell responses to ß-cell antigens in these mice after MHV68-EGFP infection, we tested their splenic T cell responses to an islet lysate from wildtype C57BL/6 mice. Since this islet lysate does not contain EGFP or MHV68 proteins, any T cell reactivity to this lysate will be against natural ß-cell antigens. We also tested for T cell responses to GAD65 since it is a fairly large protein that should contain many determinants presented by H-2^b^. As a positive control, we tested for MHV68-EGFP-induced T cell responses to EGFP. Because highly purified endotoxin-free EGFP is not commercially available, we obtained transgenic C57BL/6 mice that have a ß-actin promoter driving EGFP expression (CAG-EGFP mice), leading to high levels of EGFP expression in their muscle and used a muscle homogenate from these mice (or control nontransgenic C57BL/6 mice) to test for induced T cell responses to EGFP.

**Figure 5:**
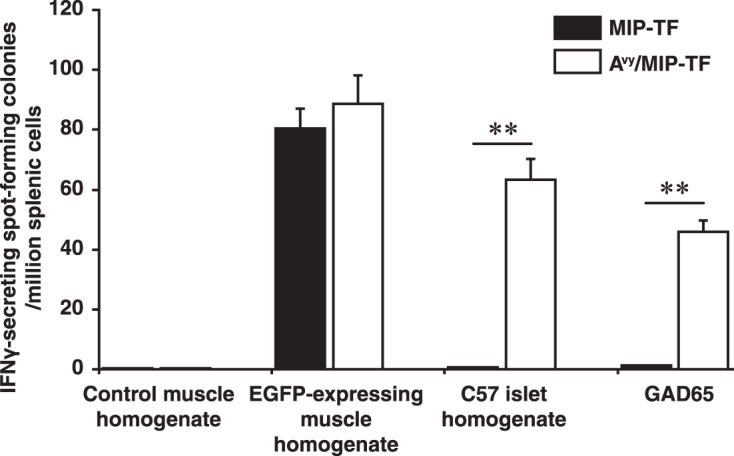
Th1-type autoimmunity spreads from EGFP to other β-cell antigens in obese, but not lean, MHV68-EGFP-infected mice. MIP-TF and A^vy^/MIP-TF mouse littermates were infected with MHV68-EGFP and 21 days later the frequency of antigen-specific IFNγ-secreting splenic T cell responses from individual mice was determined by ELISPOT assay. Data are expressed as mean IFN_γ_-secreting spot forming colonies (SFC) per million mononuclear cells ± SEM. N = 5 mice/group. The frequency of IFNγ-secreting SFC in response to control muscle homogenate (by both MIP-TF and A^VY^/MIP-TF spleen cells) and to GAD65 by MIP-TF spleen cells were near to the base-line. Splenic cells cultured at medium alone resulted in ≤5 SFC. **p < 0.01.

We infected MIP-TF and A^vy^/MIP-TF littermate mice with MHV68-EGFP and twenty-one days later tested splenic mononuclear cells from individual mice for the frequency of IFNγ-secreting spot forming colonies to a panel of antigens by ELISPOT. None of the mice displayed T cell responses to the control wildtype C57BL/6 muscle homogenate (Fig. 5). Both MIP-TF and A^vy^/MIP-TF mice infected with MHV68-EGFP displayed frequent IFNγ-secreting responses to muscle homogenate from CAG-EGFP mice. Thus, MHV68-EGFP infection induced strong immune responses to EGFP that were of a similar magnitude in both transgenic mouse models. Evidently, the A^vy^ polymorphism does not affect the magnitude of the immune response to MHV68-EGFP. Importantly, the immune response to islet lysate was at background levels in MHV68-EGFP-infected MIP-TF mice, indicating that the induced Th1 response to EGFP did not spread to other ß-cell antigens. In contrast, frequent Th1 responses to islet lysate were observed in infected A^vy^/MIP-TF mice (Fig. 5). The response to islet lysate is likely to represent only a small portion of the total T cell autoreactivity to many different ß-cells antigens because most ß-cell antigens within the lysate were not at their optimal concentrations for the ELISPOT assay. Paralleling the responses to islet lysate, the MHV68-EGFP induced Th1 response to EGFP failed to spread to GAD65 in MIP-TF mice, but led to fairly frequent Th1 responses to GAD65 in A^vy^/MIP-TF mice. Thus, in A^vy^/MIP-TF mice the response to EGFP caused bystander activation of naive GAD65-reactive T cells and drove them toward a proinflammatory phenotype. The spreading of autoreactivity among ß-cell antigens was associated with the transition from peri-insulitis to intra-insulitis.

## Discussion

Previous epidemiological studies noted an association between weight and increased risk of ß-cell autoantibodies and subsequent progression to T1D, while others have not (e.g.,)^9–14^. It is conceivable that obesity may potentiate spontaneous T cell autoreactivity to ß-cells, but these responses go unnoticed in the vast majority of individuals because the autoreactive T cells have a very low frequency and do not modulate ß-cell autoantibody seroconversion or cause T1D due to immunoregulation, anergy, exhaustion, and/or protective genetic factors. Here, we experimentally tested whether obesity could increase the propensity for developing T cell autoreactivity to ß-cells in mouse models.

Our studies demonstrated that infection with MHV68-EGFP, but not wildtype MHV68, infection induced low-grade peri-insulitis in the islets of MIP-GFP and MIP-TF mice, but not wild type mice. MHV68 is closely related to human Epstein-Barr virus and Kaposi sarcoma-associated herpesvirus and can establish persistent lifelong infections. MHV68 replicates primarily in the lung epithelium, intestine and liver and no active or latent virus is detectable in their pancreas^37,38^. Infection with wildtype MHV68 delays the development of T1D in NOD mice^38^. Since MHV68-EGFP expresses EGFP constitutively from a CMV promoter, it is expected to drive the long-term expression of EGFP in recipients.

Our observations are consistent with the notion that viral expression of EGFP induces a low-grade autoreactivity to EGFP-expressing ß-cells. The findings have similarities to previous studies of LCMV transgenic mouse models and low-dose STZ models that have provided key insights into autoimmune mechanisms^19–22^, but they differ from these past studies because: (1) the LCMV models cause acute and irreversible ß-cell destruction. In contrast, MHV68 is less cytolytic and establishes a life-long persistent infection. (2) The low-dose STZ model relies on the toxicity of STZ; it does not provide a model of obesity-related metabolic stress or chronic systemic inflammation. (3) Previous studies did not examine the influence of chronic obesity.

We next generated obese A^vy^/MIP-TF mice and found that infecting these mice at a young age (10–12 weeks old, soon after they had developed obesity) with MHV68-EGFP, but not wildtype MHV68, lead to some intra-insulitis. The infiltrates were comprised of CD3^+^ lymphocytes, macrophages and B cells, as occurs in spontaneously diabetic NOD mice. MHV68-EGFP infection of older A^vy^/MIP-TF mice (25–30 weeks old) that had been severely obese for a longer period lead to a more severe intra-insulitis.

The inability of MHV68-EGFP infection to induce a more pathogenic autoimmune response against EGFP-expressing ß-cells may be because: (1) MHV68 is not very cytolytic; (2) EGFP is not very immunogenic in wildtype C57BL/6 mice^39^ and should be even less immunogenic in MIP-TF mice in which it is a self-antigen that can induce tolerance; (3) C57BL/6 mice carry T1D-resistant MHC alleles; (4) there are insufficient co-stimulatory signals; (5) the experimentally-induced autoreactive T cells were controlled by natural regulatory responses; and/or (6) the continual production of EGFP by MHV68-EGFP-infected cells in the absence of ß-cell destruction may have led to regulatory responses to EGFP that limited ß-cell damage^40^.

Interestingly, MHV68-EGFP infection induced similar magnitude T cell responses to EGFP in both lean MIP-TF mice and obese A^vy^/MIP-TF mice, but in A^vy^/MIP-TF mice, the Th1 autoimmunity to EGFP spread to other ß-cell antigens. The spreading of T cell autoreactivity among ß-cell antigens was associated with the transition from peri-insulitis (in infected MIP-TF mice) to intra-insulitis (in A^vy^/MIP-TF mice) and may have been a driving force for intra-insulitis development.

Our observations are consistent with the notion that the obesity-associated chronic low-grade systemic inflammation provides immuno-stimulatory signals (such as circulating inflammatory factors, altered ß-cell antigen presentation, activated APC, inflammatory cytokines/chemokines in the islets and/or pancreatic lymph node, and/or reduced regulatory responses) which enable T cells recognizing other ß-cell antigens to receive sufficient co-stimulation to become activated and expand. In some individuals with a susceptible genotype and environmental exposure history, this could lead to ß-cell damage, increased ß-cell antigen presentation and eventual progression to T1D. This scenario emphasizes the role of obesity-associated systemic inflammatory factors and/or ß-cell stress in lowering the threshold needed for T cell interactions with self-MHC/peptide to result in T cell activation and expansion. This model is a modification of the accelerator hypothesis whose emphasis is on increasing body mass causing insulin resistance and glucotoxicity, which accelerates ß-cell apoptosis leading to increased presentation of ß-cell antigens^3,4^.

Other scenarios are also possible. Although the A^vy^ gene has not been reported to be expressed in immune cells or ß-cells, it is possible that low-level ectopic expression of the A^vy^ gene product in these cells could have altered immune cell function or ß-cell antigen presentation in ways that enhanced ß-cell autoreactivity independently of obesity. However, the magnitude of the Th1 responses evoked by MHV68-EGFP were of similar magnitude in both MIP-TF and A^vy^/MIP-TF mice, and immune cells from A^vy^ mice have been noted to have reduced responses against grafts^41^. Even if A^vy^-linked alterations in immune cell function or ß-cell antigen presentation contributed to our observations, these factors also contribute to T1D susceptibility within the human population, such that our results still reflect how obesity may increase the risk for ß-cell autoreactivity in some individuals.

## Methods

### Animals

All procedures involving animals were conducted in accordance with the Guide for the Care and Use of Laboratory Animals (Washington, DC, USA) and were approved by UCLA’s Animal Research Committee. Transgenic C57/BL6 mice that express GFP under the control of a mouse insulin promoter (“MIP-GFP” mice)^23^ were generously provided by Manami Hara (University of Chicago). We previously described the generation of C57/BL6 MIP-TF mice that have a mouse insulin promoter linked to a trifusion of EGFP, luciferase and HSV1-sr39TK cDNAs that are linked together to form one trifusion reporter protein. These mice express the fusion protein specifically in their ß-cells and have normal responses to intraperitoneal glucose challenge^24^. The mice are available through the Jackson Laboratory (catalog #12943).

MIP-TF mice that were heterozygotic for the transgene were mated with C57BL/6-A^vy^ mice that were heterozygotic for the agouti viable yellow (A^vy^) polymorphism to obtain wildtype, MIP-TF, A^vy^ and A^vy^/MIP-TF C57BL/6 mice. Littermates were used for each study. Offspring were genotyped for the presence of the MIP-TF transgene as previously described^24^ and the presence of the A^vy^ gene was indicated by their yellow coat color. Transgenic C57BL/6 mice that have a chicken ß-actin promoter and cytomegalovirus (CMV) enhancer driving widespread EGFP mice expression (“CAG-EGFP” mice) were obtained from the Jackson Laboratory.

### Diets

The mice were housed in microisolator caging and provided normal chow (Lab Research Diets, Catalog 5001) or high fat chow containing 60% fat (Research Diets, D12492) as indicated. Food and water were available ad libitum at all times.

### Viruses and vaccinations

#### Adenovirus

A stock of adenovirus expressing EGFP (Adeno-EGFP) was generously supplied by Dr. Sanjiv Sam Gambhir (Stanford University). Mice received 1 × 10^6^ plaque forming units (PFU) of Adeno-EGFP intravenously through their tail vein.

#### LCMV

LCMV expressing GFP (LCMV-GFP) was a generous gift from Dr. de la Torre (Scripps Research Institute, La Jolla). The mice were inoculated with 1 × 10^6^ PFU of LCMV-GFP intraperitoneally.

#### Mouse herpes virus

The construction and production of MHV68 expressing EGFP (MHV68-EGFP) viral stock has been previously described^25,26^. This virus drives the constitutive expression of EGFP from a CMV promoter. Mice were infected either at 10–12 weeks in age, or 25–30 weeks in age (as indicated) intraperitoneally with 1 × 10^6^ PFU of MHV68-EGFP or control wildtype MHV68, as per our previous studies^25,26^. Virally infected mice were monitored for the development of hyperglycemia and their pancreases were examined at the indicated time points for insulitis.

### Antigens

GAD65 was obtained from Diamyd Medical (Stockholm, Sweden). Islet lysate was prepared from isolated C57BL/6 mouse islets by sonication in sterile saline. T cell responses to EGFP were assessed using muscle tissue homogenates from CAG-EGFP C57BL/6 transgenic mice (or control wildtype C57BL/6 mice) that had been homogenized in saline, centrifuged, and the supernatant collected and frozen at −80 °C.

### ELISPOT

Twenty-one days after viral infection splenic mononuclear cells from individual mice were assayed in duplicate for IFNγ responses to GAD65 (100 μg/ml), wildtype C57BL/6 islet lysate (100 μg/well), or EGFP (100 μg CAG-EGFP muscle lysate/well) by ELISPOT in two independent studies (with 5 mice/group per study), as previously described^42,43^.

### Analysis of blood glucose, insulitis, and immune cell infiltrates

Mouse blood glucose was monitored using a One Touch Ultra Blood Glucose Monitoring System (Johnson and Johnson, CA). Diabetes onset was defined as two consecutive daily blood glucose levels >250 mg/dL.

To assess insulitis, pancreases were fixed in 10% formalin overnight sectioned at 5 µm, with every 10^th^ section histochemically stained (H&E) and examined for insulitis. Insulitis was scored using the following criteria: 0-No infiltration; 1-Some peri-insulitis; 2-Heavy perisultis with some intra-insulitis; 3-Heavy intra-insulitis. A total of 50–80 islets from each mouse were examined.

For immunohistochemical analysis of islet infiltrates, pancreata were dissected out at the indicated time point after viral infection and fixed in 10% formalin overnight. Fixed pancreatic sections (5 μm) were hydrated, treated with 3% H_2_O_2_ in methanol for 30 minutes and then subjected to antigen retrieval. Subsequently, consecutive sections were incubated with monoclonal antibodies against CD3, Mac1, CD19 or the appropriate isotype control. The bound antibodies were visualized with ABC and DAB substrate and imaged under a light microscope.

### Bioluminescence Imaging

The mice were scanned using a CCD device, as previously described^44^.

## Data Availability

All data generated or analyzed during this study are included in this published article.
